# Characterization of time of flight and resolution modeling on image quality in positron emission tomography

**DOI:** 10.1002/acm2.13751

**Published:** 2022-08-17

**Authors:** Terrance J. Moretti, Stephanie M. Leon, Colin J Schaeffer, Manuel Arreola

**Affiliations:** ^1^ Department of Radiology Division of Medical Physics University of Florida College of Medicine Gainesville Florida USA

**Keywords:** image quality, positron emission tomography (PET), resolution modelling, time of flight

## Abstract

Time‐of‐flight (TOF) and resolution modeling (RM) algorithms are frequently used in clinical PET images, and inclusion of these corrections should measurably improve image quality. We quantified the effects of these correction algorithms on reconstructed images via the following metrics: recovery coefficients (RCs), contrast‐to‐noise ratio (CNR), noise‐power spectrum (NPS), modulation transfer function (MTF), and the full width at half maximum (FWHM) of a point source. The goal of this experiment was to assess the effects of the correction algorithms when applied singly or together. Two different phantom tests were performed and analyzed by custom software. FWHM and MTF were measured using capillary tube point sources, while RCs, CNR, and NPS were measured using an image quality body phantom. Images were reconstructed with both TOF and RM, only TOF, only RM, or neither correction. The remaining reconstruction parameters used the standard clinical protocol. RM improved RCs, FWHM, and MTF, without increasing overall noise significantly. TOF improves CNR for small objects FWHM or MTF but did not decrease noise. RCs were not statistically improved by enabling these algorithms. Inclusion of both correction algorithms in image reconstruction provides an overall improvement to all metrics relative to the uncorrected image, but not by a significant margin in multiple aspects.

## 1 INTRODUCTION

Time‐of‐flight (TOF) and resolution modeling (RM) algorithms are common on modern positron emission tomography (PET) systems. When TOF is enabled, we expect recovered contrast and noise characteristics to be improved, as the location of annihilation events is known to lie within a probability distribution along the line of response. The goal of utilizing RM algorithms is to improve system spatial resolution by modeling the imaging signal chain and introducing a convolution kernel into the reconstruction of the images with information obtained from a system's measured point spread function (PSF), either within the image or sinogram domains, in order to counteract blurring.^1^ Introducing this convolution, however, may affect other aspects of image quality indirectly; in particular, PSF modeling is expected to show some frequency‐dependent impact on noise characteristics.

When enabled, TOF and RM are designed to improve image quality, yet a thorough, independent characterization of these technologies is lacking. Previous work has assessed the effects of Siemens’ correction algorithms on image quality using the National Electrical Manufacturer's Association (NEMA)‐specified metrics of percent contrast, percent background variability, and attenuation correction error.^2^, ^3^, ^4^, ^5^ Other work has been primarily qualitative in nature.^6^, ^7^ Our proposed image quality analysis also included measures such as contrast recovery coefficient (RC), contrast‐to‐noise ratio (CNR), noise power spectrum (NPS), modulation transfer function (MTF), and the full width at half maximum (FWHM) of the profile of a point source. These quantities are more descriptive of overall image quality than those previously measured. While some of these quantities have been assessed on other systems,^3^, ^5^, ^8^, ^9^, ^10^, ^11^ the current literature contains neither a collection of all the proposed measurements nor a full characterization of the Siemens system tested here. By directly comparing these quantities for reconstructions that do and do not use TOF and RM, we can better assess the potential impacts of these technologies on image quality.

Our goal in this study was to quantify the effects of one manufacturer's algorithms by use of a comprehensive, quantitative, phantom‐based image quality assessment using clinically‐relevant reconstruction protocols. We report RC, CNR, NPS, and MTF for protocols processed with and without TOF and RM. To make the metrics easy to acquire, we designed a code to gather all metrics from scans of the commonly available NEMA image quality phantom and a simple set of point sources.

## 2 METHODS

All scans were performed on a Siemens Biograph mCT Flow (Siemens Healthcare, Erlangen, Germany) running Syngo software version VG62B. The scanner uses lutetium oxyorthosilicate crystal arranged in a 13 × 13 array (size of 4 mm × 4 mm × 20 mm) per each of the 48 detector blocks. There are four detector rings, with a diameter of 84.2 cm. The bore size is 78 cm and provides an axial field of view (FOV) of 22 cm.^12^ The scanner has TOF capabilities, as well as an RM algorithm called TrueX. The system has a timing resolution of 0.55 ns and a coincidence window of 4.066 ns.^12^

Two modified phantom tests based on quality assurance tests described by the American Association of Physicists in Medicine (AAPM) and NEMA were performed. Specifically, the scan protocols used followed procedures as described in Section 4 of AAPM Task Group Report 126(TG‐126) and in Section 7 of NEMA NU‐2.^4^, ^13^ The former evaluates spatial resolution via imaging of point sources, and the latter evaluates image quality using the NEMA PET body phantom. RC, CNR, and NPS were calculated using scans of the NEMA phantom; the MTF and FWHM were calculated from imaging of point sources created using capillary tubes. The implementation of these tests is described in more detail in the following sections. All scans used F‐18 fluorodioxyglucose.

Reconstructions were generated with RM alone, with TOF alone, with both corrections together (RM + TOF), and with neither correction (NC). All scans used CT‐based attenuation correction and relative scatter scaling model‐based scatter correction. Ordered‐subset expectation maximization 3D iterative reconstruction was used; all reconstructions used two iterations and an 8 mm Gaussian postfilter. When TOF was enabled, 21 subsets were used, but this number was unavailable when TOF was disabled, so 24 subsets (the closest allowed value) were used when TOF was not active. Three separate scans were performed on the NEMA image quality phantom. The TG‐126 spatial resolution test was performed twice. NEMA phantom images were reconstructed with a matrix size of 200 × 200, while point source images were reconstructed with a matrix size of 512 × 512. Analysis of all images used custom software generated in MATLAB (Mathworks, Natick, MA).

### 2.1 NEMA phantom quantities

Phantom filling and scanning were performed in accordance with NEMA NU‐2 Section 7, excluding the additional scatter phantom, which was not used.^4^ Contrast spheres were filled with an activity concentration of 40 kBq/ml. For the 8:1 contrast ratio, the background cavity was filled with approximately 1.4 mCi, which gave an activity concentration one‐eighth of that used in the spheres. After the 8:1 scan was completed, the activity of the background was doubled (i.e., for a total of approximately 2.8 mCi) to create a 4:1 contrast ratio, and the scans were repeated. All clinically used protocols on the scanner have continuous bed motion; a common clinical protocol using a table speed of 1.4 mm/s was selected for the 8:1 concentration ratio. The table speed was doubled to 2.8 mm/s for the 4:1 scans to ensure a similar number of detected counts.

**FIGURE 1:**
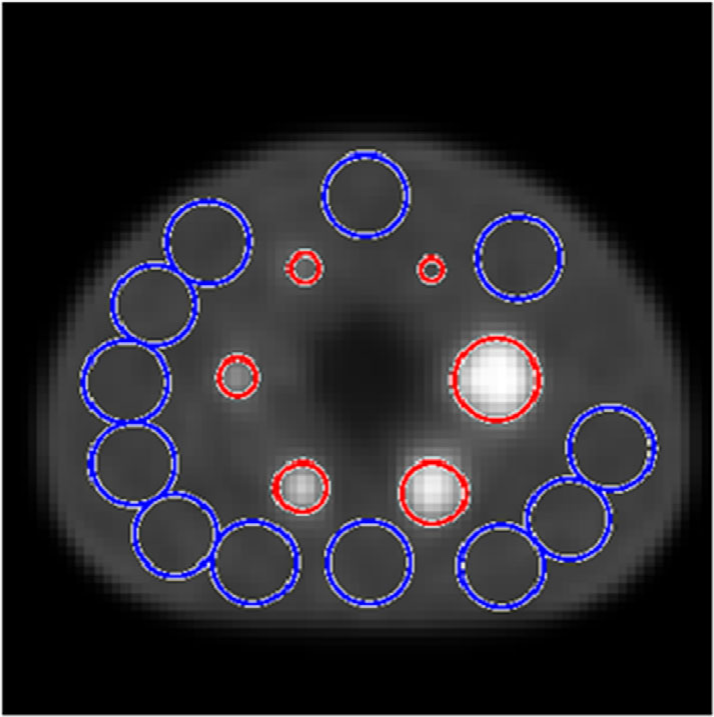
Example ROI map created when performing the National Electrical Manufacturer's Association (NEMA) image quality test. The smaller red circles indicate the six hot spheres; the larger blue circles indicate the background ROIs. Each of the 37‐mm diameter blue ROIs contains five smaller concentric ROIs with sizes matching the red ROIs, which are not shown.

The NEMA‐specified region of interest (ROI) locations were used for our measurements. Figure 1 shows where the ROIs were placed on the central slice on the phantom, with the red ROIs matched in physical size and placed at the center of the hot contrast spheres whose RC and CNR values are presented, while the blue ROIs represent the background areas which we utilized in calculation of the background for CNR and NPS.

Recovery coefficients were calculated via Equation 1:

(1) $${\mathrm{RC}}_{j}=\;\left(H_{\mathrm{measured},j}-B_{\mathrm{measured},j}\right)/\left(H_{\mathrm{true}}-B_{\mathrm{true}}\right)$$

Where RC_j_ is the recovery coefficient for hot sphere of radius j, H_measured,j_ is the mean activity concentration for the ROI centered on the hot sphere of radius j, B_measured,j_ is the global mean activity concentration for the 12 background ROIs of radius j, and H_true_ and B_true_ are the true, known activity concentrations in the hot spheres or the background, respectively.

The uniform background region of the NEMA phantom was used for the NPS calculation. From the NPS, we may determine the frequency distribution of the noise within an image, given the constraint that there is inter‐voxel correlation due to the nature of tomographic reconstruction. The NPS was determined by adapting a method from work by Friedman et al.^14^ Sixty 9‐pixel × 9‐pixel square ROIs were placed at the locations of the background ROIs used (Figure 1) in the NEMA test across five slices: the axial slice located at the center of the sphere ring and ±1 and ±2 cm from the original slice. These slices are the same ones used in the background variability calculation by the NEMA protocol. ROI size was limited to 9 × 9 to avoid the inclusion of pixels near or at the edges of the uniform region. These ROIs were then zero‐padded to 64 × 64 during the Fourier transform to increase the number of frequency bins available. The 2D NPS for the k‐th ROI is calculated via Equation 2:

(2) $${\mathrm{NPS}}_{k}\left(S_{x},S_{y}\right)\;=\;\frac{p^{2}}{N_{s}^{2}}{\left|\left[FT\left(R{\left(x,y\right)}_{k}-\bar{R_{k}}\right)\right]\right|}^{2}$$

Where *p* is the pixel size, $N_{s}^{2}$ is the matrix size, $R{\left(x,y\right)}_{k}$ is the matrix of values contained within the k‐th ROI, and $\bar{R_{k}}$ is the mean value in the k‐th ROI. The NPS of the image was determined by averaging the NPS values across all 60 square ROIs and binning the results by spatial frequency. NPS was then normalized by activity concentration in the background region. Area under the curve (AUC) values, which reflect the total noise content in the region, were calculated for each normalized NPS.

CNR combines contrast and noise together into a single metric, which may provide more insight to the inherent detectability of objects than either the RC or NPS on their own. The NEMA ROI placements were used to calculate CNR using Equation 3:

(3) $${\mathrm{CNR}}_{i}=\frac{1}{12}\;\sum_{j\;=\;1}^{12}\frac{S_{\mathrm{hot}}^{i}-S_{\mathrm{bg},j}^{i}}{\sigma_{\mathrm{bg},j}^{\left[37\right]}}$$

Where $S_{\mathrm{hot}}^{i}$ is the mean value in the ROI for hot sphere *i*, $S_{\mathrm{bg},j}^{i}$ is the mean value in background ROI *j* on the same slice as the hot object with diameter matched to the size of hot object *i*, and $\sigma_{\mathrm{bg},j}^{\left[37\right]}$ is the standard deviation (SD) of the same background ROI *j*. The use of global mean values for calculation of CNR was used to remove as much statistical noise in the results as possible. Upon initial inspection, SD values across the uniform region showed considerable variation, despite relative constancy in average signal across these regions, which dominated the CNR. Therefore, a net CNR was determined by averaging across all 12 CNR values obtained using each ROI. Use of a global background signal is used in calculation of percent contrast in NEMA NU‐2, so using this method to calculate CNR is a logical extension.^4^

### 2.2 Point source quantities

Spatial resolution and MTF measurements were obtained by following the procedures outlined in the TG‐126 spatial resolution test to create and scan point sources.^13^ We performed this spatial resolution analysis at the center of the axial FOV only. Five million counts were used for each image set.

Using axial reconstructions, FWHM values were calculated for each of the three sources’ line profiles along the tangential and radial directions of each source. While the spatial resolution values obtained this way do not reflect true clinical resolutions because the lack of a scattering medium affects the way iterative reconstruction converges, they can be used comparatively to assess the effects of correction algorithms on the same scanner.^13^

MTF was also measured using these point source images. The 2‐D MTF was calculated by obtaining the Fourier transform of a 50 mm × 50 mm square ROI around the center point source after normalization, as shown in Equation 4.

(4) $$\mathrm{MTF}\left(s_{x},s_{y}\right)\;=\left|\frac{\mathrm{FT}\left\{\mathrm{PSF}\left(x,y\right)\right\}}{\int \;\;\;\int \mathrm{PSF}\left(x,y\right)dxdy}\right|$$

A 1‐D MTF_PSF_ was approximated from the 2‐D MTF as the average of the MTF at each point along the two zero frequency lines in *s_x_* and *s_y_*. This one‐dimensional average is the reported MTF.

### 2.3 Statistical analysis

Statistical analysis was performed with JMP Pro (SAS Institute Inc, Cary, NC). A *p*‐value less than 0.05 was considered significant. Analysis of datapoints for RC, CNR, and NPS AUC used the Friedman test, as to address multiple repeated measures comparisons. Any results to have a significant difference using Freidman analysis were then tested post hoc using Dunn's test. Differences in MTF were assessed via Tukey's test for the differences across all points in frequency space. Paired *t*‐tests were used to determine the effects on FWHM from different corrections.

## 3 RESULTS

### 3.1 Recovery coefficients

**FIGURE 2:**
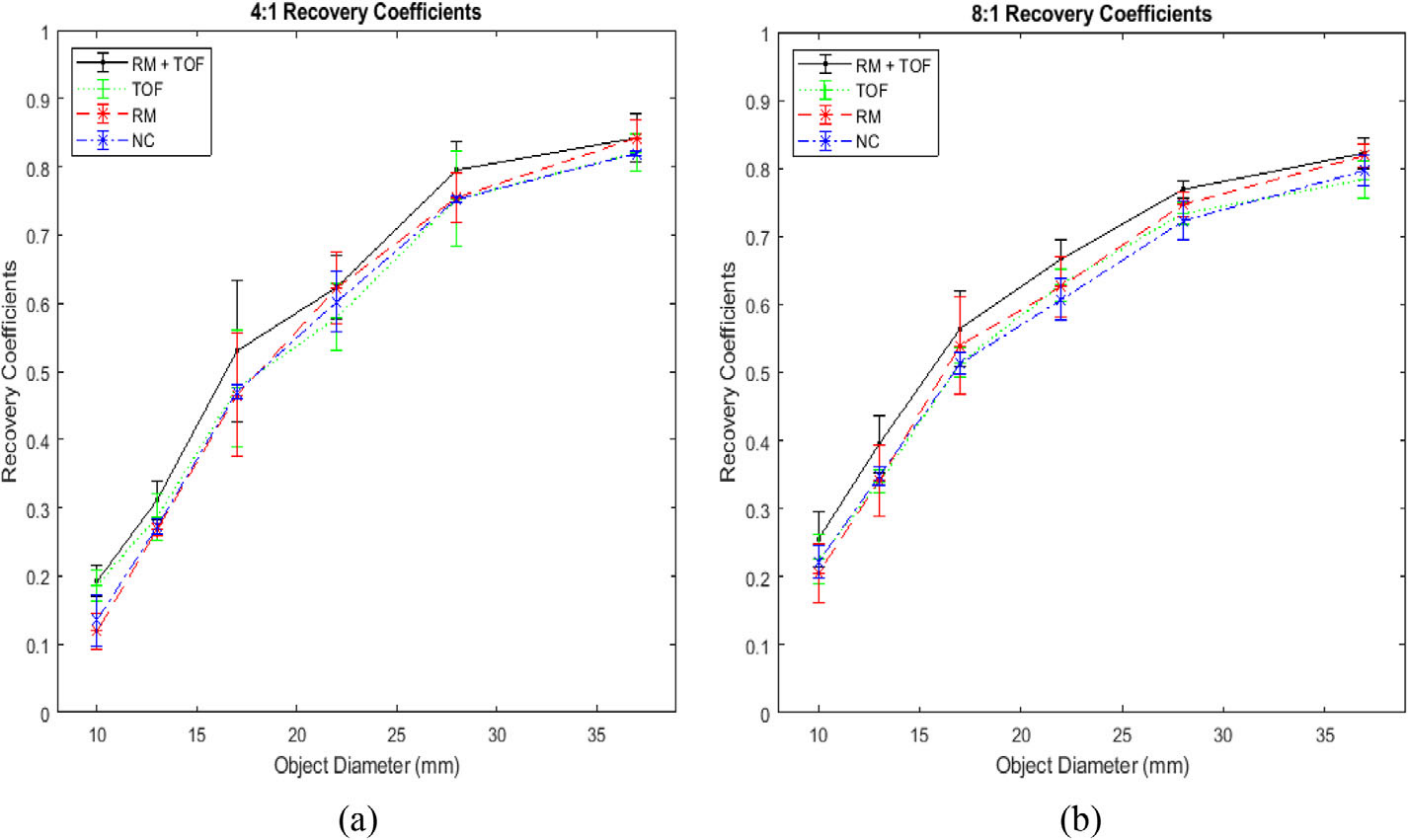
Recovery coefficents for (a) 4:1 sphere‐to‐background activity ratio and (b) 8:1 sphere‐to‐background activity ratio. Error bars represent standard deviations. Enabling both resolution modeling (RM) and Time‐of‐flight (TOF) appears to provide the best recovery coefficient (RC) value for all object sizes, but this difference was not significant.

RCs for the contrast spheres at both activity ratios are shown in Figure 2. Overall differences were minor, but some trends can be observed. In the 4:1 case, Friedman's test revealed a significant difference between the means for the smallest object size (*p* = 0.0421), but Dunn's test revealed no significant differences for any individual pairs. RM slightly, but not significantly, appears to improve the recovery coefficients for the largest objects; this is not true for the smallest objects, where the NC images recover similar or more contrast than the RM reconstructions. For the smallest spheres in the 4:1 images (Figure 2a), the reconstructions with only RM appear to recover less contrast relative to those with TOF, while in the 8:1 ratio (Figure 2b), the TOF RCs are about equal to the NC and RM RCs. Enabling both RM + TOF appears to provide the highest mean values of RCs across object sizes, regardless of activity ratio. However, statistical analysis showed no significant differences between any correction algorithm recovery coefficient values for all object sizes across both ratios.

### 3.2 NPS

**FIGURE 3:**
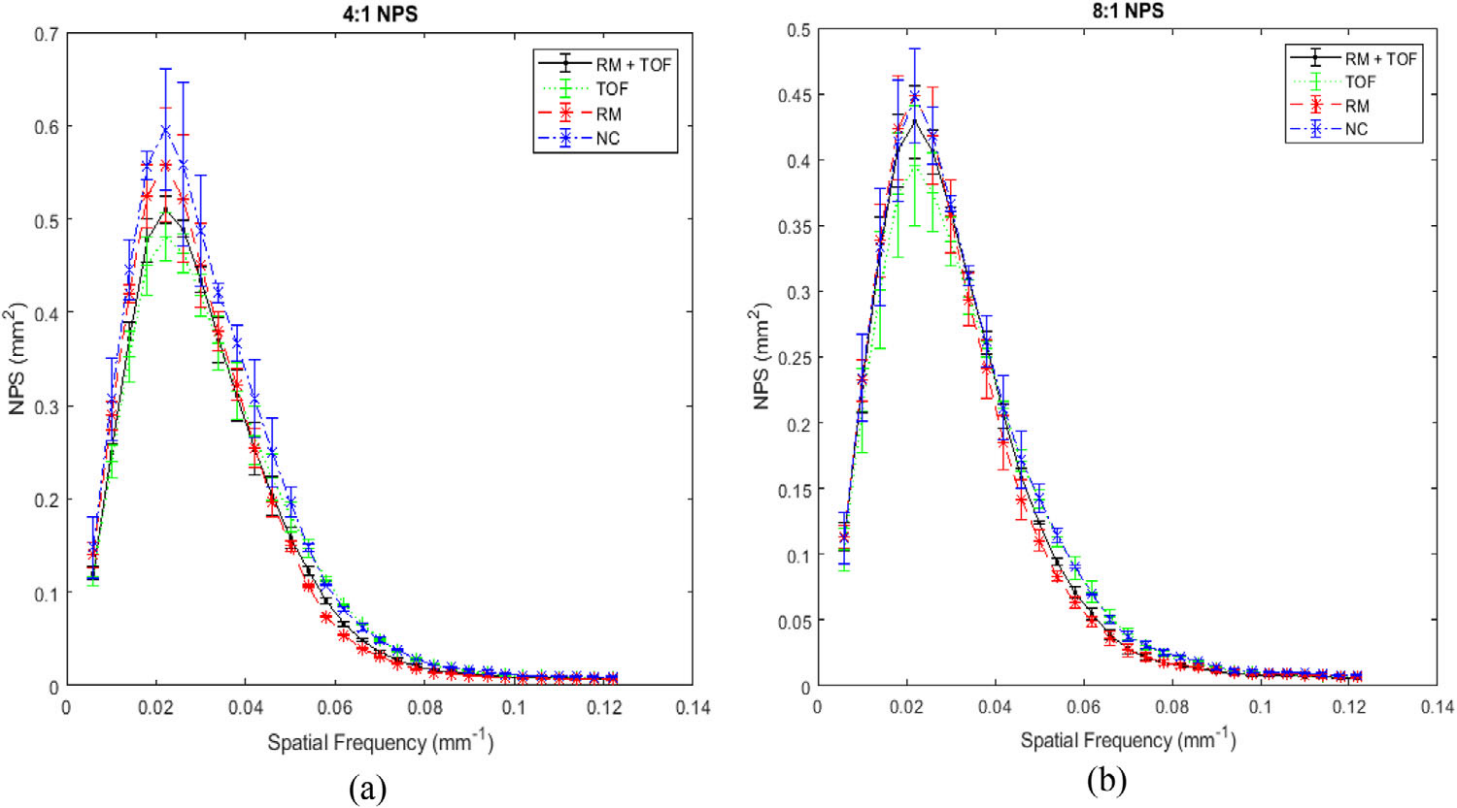
Noise‐power spectrum (NPS) curves obtained for the (a) 4:1 and (b) 8:1 activity ratios. Curves have been normalized to the activity present at scan time. Error bars represent standard deviations.

Figure 3 shows the NPS curves for both activity ratios, normalized by activity at scan time. The peak noise frequency of the NPS did not change across correction algorithms. For low spatial frequencies, inclusion of TOF decreased the noise magnitude, while inclusion of RM increased noise magnitude. Interestingly, this trend seems to flip at higher frequencies; the TOF‐only protocol transitions from lowest relative noise at low frequencies to higher or highest relative noise at higher frequencies, while the RM‐only protocol shows the lowest relative noise at higher frequencies. NC images show high noise at most frequencies. Use of both TOF and RM together provided noise levels in between TOF‐alone and RM‐alone, for both the lower and higher frequencies.

NPS AUC values are shown in Table 1. These AUC values provide a numerical estimate of total noise in an image. The NC protocol has the highest noise magnitude in both activity ratios, while the three corrected protocols have similar AUC values to one another. Differences between all AUC values are small, and, in the 8:1 case, the TOF protocol is within one SD of the NC protocol. No significant differences between any reconstruction methods for the 4:1 (*p* = 0.071) nor the 8:1 ratio (*p* = 0.284) were found, implying that total noise content is unaffected by the choice of correction algorithm.

**TABLE 1:** Nois power spectrum (NPS) area under the curve (AUC) values

RM enabled	TOF enabled	4:1 AUC ± SD (mm)	8:1 AUC ± SD (mm)
X	X	0.0176 ± 0.0005	0.0148 ± 0.0006
X		0.0183 ± 0.0008	0.0146 ± 0.0007
	X	0.0179 ± 0.0009	0.0146 ± 0.0011
		0.0209 ± 0.0000	0.0157 ± 0.0003

Abbreviations: RM, resolution modeling; SD, standard deviation; TOF, time‐of‐flight.

### 3.3 CNR

**FIGURE 4:**
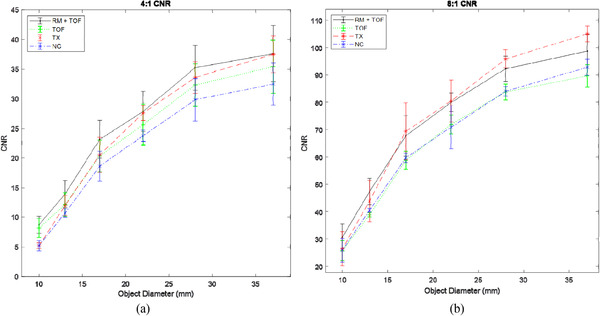
Contrast‐to‐noise ratio (CNR) plots for (a) 4:1 sphere to background ratio and (b) 8:1 sphere to background ratio. Error bars represent standard deviations.

CNR behavior trends similarly to RC curves, but some differences are apparent (Figure 4). Friedman's test showed a significant difference for the 10 mm sphere only in the 4:1 ratio (*p* = 0.0421), but Dunn's test revealed no significant differences between pairs. In the 8:1 ratio, CNR values was found to be different for the 37‐mm and 28‐mm spheres (*p* = 0.0421 in both cases), but no statistical difference was found between any protocols for the smaller objects.

### 3.4 MTF and resolution

**FIGURE 5:**
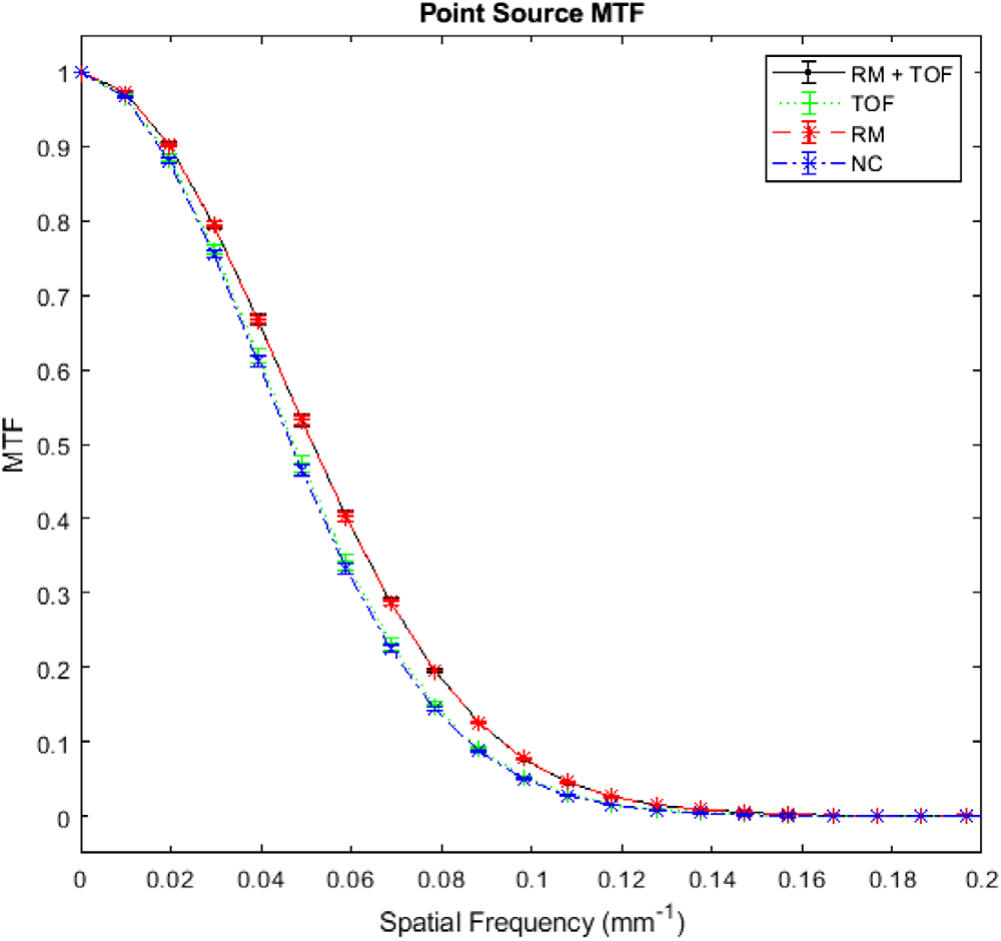
Modulation transfer function (MTF) curves obtained from the point source images. Error bars represent standard deviations. Improvements in MTF when using resolution modeling (RM) are shown, and the independence from Time‐of‐flight (TOF) is illustrated.

MTFs were found to be significantly different overall (*p* < 0.0001). MTF curves are shown in Figure 5. Including RM improved MTF relative to the NC protocol (*p* = 0.0007) and the TOF‐only protocol. Using both RM + TOF did not significantly increase MTF once RM was already applied (*p* = 0.136), but TOF did improve MTF over the NC protocol (*p* = 0.0009), which is difficult to glean from Figure 5. For these MTF curves, limiting resolutions (where MTF = 0.1) are approximately 0.086 mm^–1^ for NC and TOF and 0.094 mm^–1^ for RM and RM + TOF.

Table 2 shows the average FWHM values with SDs for differing reconstruction parameters. There was a significant difference between FWHM values for reconstructions with RM compared to those without RM (*p* < 0.001), and no significant difference for reconstructions with TOF compared to those without TOF (*p* = 0.921). The RM resolution recovery algorithm was able to improve the spatial resolution by greater than 0.5 mm in both the tangential and radial directions.

**TABLE 2:** Full width at half‐maximum (FWHM) values from point sources

RM enabled	TOF enabled	Average radial FWHM ± SD (mm)	Average tangential FWHM ± SD (mm)
X	X	8.55 ± 0.08	8.63 ± 0.07
X		8.52 ± 0.06	8.58 ± 0.08
	X	9.31 ± 0.10	9.41 ± 0.22
		9.36 ± 0.07	9.21 ± 0.03

Abbreviations: RM, resolution modeling; SD, standard deviation; TOF, time‐of‐flight.

## 4 DISCUSSION

Overall, RM and TOF affect different image quality quantities in different ways, with exact effects being dependent on object size and activity, but many of these effects are small and were found not to be statistically significant. However, some trends can be seen in the data.

The improvements in TOF‐enabled CNR at the lower activity ratio are not unexpected, as TOF is known to increase signal‐to‐noise ratio (SNR) when used in reconstructions.^11^ This could also help to explain the improvement in MTF over the NC protocol. However, this improvement seems to reach a plateau, as in the higher activity ratio, TOF does not improve CNR compared to the NC protocol, even for the smallest objects. It should be noted that the expected SNR gain from TOF is relatively small considering the 550 ps timing resolution of the system and the limited size of the phantom, which likely contributed to these nonsignificant results using TOF at higher activity ratios, which already have a high SNR. In the clinical scenario, however, it is likely that many smaller lesions may have low differential uptake. TOF's increases in CNR should result in improvements in detectability for these low‐contrast lesions in vivo, which is desirable.

NPS AUCs show that the reconstructions are roughly equal in overall noise, which was unexpected. Initially, we expected RM to increase noise and TOF to decrease noise, but neither was strictly true. Findings in the existing literature are inconsistent. Two previous studies found that RM decreased the noise coefficient of variation or background variability for most objects of similar sizes to those presented in this work.^10^, ^11^ However, Rahmim and Tang found that RM not only increases magnitude of the NPS but also shifts the frequency distribution.^8^ Previous literature has suggested that TOF reduces background variability, but only for large numbers of iterations.^3^, ^5^ Noise characteristics are expected to change with more iterations, but the number of iterations used in many prior works do not reflect those used clinically at our institution and thus are of questionable relevance. More work may be necessary to fully describe the effects of these correction algorithms on image noise.

Noise texture may play an important role in driving protocol selection. RM and TOF may not significantly alter total noise, but qualitatively, they do appear to shift the relative noise distributions to a small degree. While the peak noise frequency did not change across reconstructions, RM shifts the relative noise magnitude slightly toward lower frequencies, while TOF shifts it slightly toward higher frequencies. Selection of a best NPS distribution, and thus noise texture, may have consequences for in vivo images.

By using both RM + TOF, RM's improvements in large object CNR may be maintained while still improving CNR for small objects with low innate contrast. The latter finding is relevant, as higher CNR for small lesions in a situation with low differential uptake is desirable, which cannot be said for the former finding. While improving CNR of large objects is not undesirable per se, those objects likely have high contrast and are thus not in need of further enhancement to be detectable.

RM improvements to both MTF and FWHM are clearly demonstrated. However, TOF's improvement of MTF is not reflected in FWHM. This improvement was significant but small in magnitude. One potential explanation could be that, per TG‐126, the point sources are imaged for 5–10 million counts, and there is no scattering medium^13^; therefore, these images are beyond some contrast plateau where TOF does not appear to improve contrast. Analysis of the MTF using more clinically representative conditions would make an interesting comparison to this ideal case of a point source in air.

In summary, RM may improve CNR for large objects in the higher activity ratio, and improves both MTF and spatial resolution. TOF appeared to shift the NPS toward higher spatial frequencies and had a small improvement to MTF while not affecting FHWM. Individual use of either RM or TOF improves image quality over not using either; when combined, the sum of their improvements may lead to optimal image quality in most cases. However, while some results were found to be significant, many other trends were not found to be significant in this experiment. RCs specifically were found not to be significantly affected by either correction algorithm, even when combined together. This raises the question of why only some data resulted in significant conclusions, but others were not. The most likely explanation is that the data are not comprehensive, as a result of insufficient sampling. Only three different scans were done using the NEMA phantom. Many of the parameters investigated are contrast‐dependent, and discrepancy between studies could be fairly large, as indicated by the SD bars in Figures 2, 3, 4. A repeat experiment with increased scan times or more collected data may find statistically significant improvements that were missed in our analysis (particularly for RC values).

General trends in our results agree with the limited results available from prior studies.^3^, ^5^, ^10^ RM increases in spatial resolution^3^, ^10^ and contrast^5^, ^10^ were well known, and TOF's minimal impact on FWHM was documented by Suljic et al. for the same system as the one in this study.^3^ However, no previous works included all of the metrics shown here. By including a more comprehensive set of image quality metrics in this work to characterize RM and TOF, we may paint a more complete picture of their effects on medical images and visualize interplay between different metrics. Our collection of metrics further shows trends not immediately apparent in prior works, such as the plateau of TOF improvements to contrast at higher activity ratios and the shifting of noise distribution in the NPS. This effect explains an apparent discrepancy between the results from Suljic et al. indicating minimal improvements to contrast recovery for small objects when using TOF in an 8:1 ratio^3^ and findings from Bettinardi et al. indicating that TOF did improve small object contrast for a 4:1 ratio in the NEMA phantom.^5^

A further limitation of this work is that all metrics examined were taken from phantom images; no in vivo assessments of image quality were performed. Including RM and TOF correction algorithms in the reconstructed images may very well lead to improvements in various quantitative metrics, but the overall diagnostic quality was not assessed. The current whole‐body protocol in use at our institution (8 mm Gaussian postfilter, 2 iterations, 21 subsets, RM and TOF enabled) provides relatively good results for most metrics, so no immediate change to our protocol was recommended as a result of this study. However, a more thorough, task‐based consideration of protocols should be performed to ensure that the best possible diagnostic image reconstructions are being provided. Regarding reconstructions, only one filter type and few selections of iterative reconstruction updates (iterations · subsets) out of many possibilities were considered. These reconstruction options likely have different noise or recovery characteristics than the selections we chose and thus have the potential to change image quality with potentially higher magnitudes than RM and TOF. Particularly, the effects of iterations on NPS structure should be investigated. Future studies using this method should be performed for more reconstruction parameters, with the eventual goal of developing a methodology to use the methods or findings presented in this work for protocol optimization.

## 5 CONCLUSION

An analysis of RM and TOF effects on image quality was performed with widely used phantoms. RC, NPS, CNR, MTF, and spatial resolution were measured for reconstruction protocols that had both RM + TOF enabled, protocols with only one or the other enabled, and protocols with neither enabled. RC values were statistically unaffected by the reconstruction algorithms. RM improves large object CNR, MTF, and spatial resolution FWHM, without increasing overall noise significantly. TOF increases small object CNR for a low activity ratio and improves MTF slightly, relative to an uncorrected protocol.

## CONFLICT OF INTEREST

The authors have no conflict of interest to disclose.

## AUTHOR CONTRIBUTIONS

*Manuscript writing, data acquisition, and all data analysis excepting noise power spectrum*: TM. *Data acquisition and manuscript revisions*: SL. *Noise power spectrum data analysis and manuscript revisions*: CS. *Project oversight and guidance and manuscript revisions*: MA.

## References

[1] Rahmim A , Qi J , Sossi V. Resolution modeling in PET imaging: theory, practice, benefits, and pitfalls. Med Phys. 2013;40(6):064301. 10.1118/1.4800806 23718620 PMC3663852

[2] Surti S. Update on time‐of‐flight PET imaging. J Nucl Med. 2015;56(1):98‐105. doi:10.2967/jnumed.114.145029 25525181 PMC4287223

[3] Suljic A , Tomse P , Jensterle L , Skrk D. The impact of reconstruction algorithms and time of flight information on PET/CT image quality. Radiol Oncol. 2015;49(3):227‐233. doi:10.1515/raon-2015-0014 26401127 PMC4577218

[4] National Electrical Manufacturers Association . NEMA Standards Publication NU 2‐2018: Performance Measurements of Positron Emission Tomographs. National Electrical Manufacturers Association ; 2018.

[5] Bettinardi V , Presotto L , Rapisarda E , Picchio M , Gianolli L , Gilardi MC . Physical performance of the new hybrid PET/CT discovery‐690. Med Phys. 2011;38:5394‐5411. doi:10.1118/1.3635220 21992359

[6] Sharifpour R , Ghafarian P , Bakhshayesh‐Karam, Jamaati H , Mohammad RA . Impact of time‐of‐flight and point‐spread‐function for respiratory artifact reduction in PET/CT imaging: focus on standardized uptake value. Tanaffos. 2017;16(2):127‐135. https://www.ncbi.nlm.nih.gov/pmc/articles/PMC5749325/ 29308077 PMC5749325

[7] Shang K , Cui B , Ma J , et al. Clinical evaluation of whole‐body oncologic PET with time‐of‐flight and point‐spread function for the hybrid PET/MR system. Eur J Radiol. 2017;93:70‐75. 10.1016/j.ejrad.2017.05.029 28668434

[8] Rahmim A , Tang J. Noise propagation in resolution modeled PET imaging and its impact on detectability. Phys Med Biol. 2013;58:6945. 10.1088/0031-9155/58/19/6945 24029682 PMC3866837

[9] Karakatsani NA , Lodge MA , Rahmim A , Zaidi H. Introducing Time‐of‐Flight and Resolution Recovery Image Reconstruction to Clinical Whole‐Body PET Parametric Imaging. IEEE; 2014. 10.1109/NSSMIC.2014.7430771

[10] Lee YS , Kim JS , Kim KM , Kang JH , Lim SM , Kim HJ. Performance measurement of PSF modeling reconstruction (True X) on Siemens Biograph TruePoint TrueV PET/CT. Ann Nucl Med. 2014;28:340–348. 10.1007/s12149-014-0815-z 24504938

[11] Akamatsu G , Ishikawa K , Mistumoto K , et al. Improvement in PET/CT image quality with a combination of point‐spread function and time‐of‐flight in relation to reconstruction parameters. J Nucl Med. 2012;53(11):1716‐1722. 10.2967/jnumed.112.103861 22952340

[12] Rausch I , Cal‐González J , Dapra D , et al. Performance evaluation of the Biograph mCT Flow PET/CT system according to the NEMA NU2‐2012 standard. EJNMMI Phys. 2015;2(1):26. 10.1186/s40658-015-0132-1 26501827 PMC4883615

[13] Mawlawi OR , Kemp BJ , Jordan DW , et al. PET/CT acceptance testing and quality assurance: the report of AAPM task group 126. 2019 10.37206/193 33320364

[14] Friedman SN , Fung GSK , Siewerdsen JH , Tsui BMW. A simple approach to measure computed tomography (CT) modulation transfer function (MTF) and noise‐power spectrum (NPS) using the American College of Radiology (ACR) accreditation phantom. Med Phys. 2013;40(5):051907. 10.1118/1.4800795 23635277 PMC3643984

